# Folic Acid Supplementation and Spontaneous Preterm Birth: Adding Grist to the Mill?

**DOI:** 10.1371/journal.pmed.1000077

**Published:** 2009-05-12

**Authors:** Leonie Callaway, Paul B. Colditz, Nicholas M. Fisk

**Affiliations:** 1 School of Medicine, The University of Queensland, Brisbane, Queensland, Australia; 2 Royal Brisbane & Women's Hospital, Herston, Queensland, Australia; 3 University of Queensland Centre for Clinical Research, The University of Queensland, Brisbane, Queensland, Australia

## Abstract

Nicholas Fisk and colleagues discuss a new study reporting that additional voluntary folic acid supplementation was associated with a major reduction in very preterm births.

**Linked Research Article**

This Perspective discusses the following new study published in *PLoS Medicine*:

Bukowski R, Malone FD, Porter FT, Nyberg DA, Comstock CH, et al. (2009) Preconceptional folate supplementation and the risk of spontaneous preterm birth: A cohort study. PLoS Med 6(5): e1000061. doi:10.1371/journal.pmed.1000061

In an analysis of a cohort of pregnant women, Radek Bukowski and colleagues describe an association between taking folic acid supplements and a reduction in the risk of preterm birth.

Preterm birth is increasing, and complicates 12% of deliveries in the United States. It is the dominant cause of neonatal mortality. Preterm birth also accounts for one in three children with vision impairment, one in five with mental retardation, and almost half with cerebral palsy [1]. Babies born weighing under 2,500 g are at heightened risk in adulthood of diabetes and cardiovascular disease [1]. These short- and long-term sequelae make the prevention of preterm birth a public health priority.

## Therapeutic Nihilism in Preterm Labor

Although some preterm births are indicated for maternal or fetal complications, most are spontaneous. Yet there is no licensed tocolytic agent available in the US to treat early-onset contractions, no treatment for threatened preterm labor that improves neonatal outcome, and no new class of drug under development [2]. In the face of such therapeutic nihilism, attention has turned instead to prophylaxis. There is encouraging evidence that prophylactic progesterone in women at increased risk (shortened cervix, previous history) reduces the incidence of very preterm birth [3],[4]. Enthusiasm for this approach has been dampened by two setbacks. First, progesterone does not work in all pregnancies at risk of preterm labor (specifically twins). Second, 17-hydroxyprogesterone caproate recently failed to win approval from the US Food and Drug Administration due to safety concerns about fetal death rates in monkeys and humans [5],[6].

## Prophylactic Folic Acid: Newfound Benefit of an Old Approach?

Poor periconceptional nutrition is implicated in idiopathic preterm labor in both animal models and human studies. As with the progesterone story, there is renewed interest in decades-old suggestions that folic acid may reduce preterm birth [7]–[9]. Because of poor compliance with recommendations to take periconceptional folate supplements to prevent neural tube defects (NTDs), more than 50 countries have already introduced mandatory wheat flour fortification [10]–[12]. In California, this was associated with a modest reduction in low birthweight and preterm birth [13].

In this issue of *PLoS Medicine*, Radek Bukowski and colleagues report that additional voluntary folic acid supplementation was associated with a major reduction in very preterm births, those most at risk of adverse outcomes [14]. Women taking supplements for at least one year before conception had a 70% reduction in spontaneous preterm birth between 20–28 weeks, and a 50% reduction between 28–32 weeks, when compared to those with no additional supplementation. Long-term compared to no supplementation was associated with a reduction in the risk of spontaneous preterm birth before 32 weeks from one in 154 to one in 423.

Given the multiple causes of preterm labor, is this degree of hazard reduction plausible [15]? Biologically, the authors point to anti-inflammatory mechanisms. On the one hand, infection is implicated in only a minority of preterm births, but on the other, infection is found increasingly at earlier gestational ages, such as those suggested to benefit most in this study. In keeping with this mechanism, no association was seen with non-infective phenomena, such as indicated preterm birth or disorders of placentation.

Methodologically, the study has several strengths, and is unlikely to be replicated. It is based on a huge dataset, with prospective recording of dietary supplements and potential confounders, and gestational age determined accurately on first trimester ultrasound. Those born preterm because of intervention were appropriately censored.

Nevertheless, there remains need for caution. This was a secondary analysis of a Down syndrome screening study, and so information on folic acid dose, formulation (with or without other supplements), and daily compliance is incomplete. In relation to numerators, there were only 16 births at under 32 weeks in those on long-term supplements. One unanswered question is whether women on folic acid for at least one year took a larger dose more diligently, resulting in higher folate levels at conception compared to women taking shorter-term supplements (women taking supplements were better educated, white, non-smoking, and different to the remaining women on nearly every measured parameter). Indeed, controlling for confounders negated the significance of any effect of less than one year's supplementation. Long-term consumption of preconceptional folic acid supplements in other countries might suggest a subfertility/planned pregnancy effect, but Bukowski's study occurred against the backdrop not only of mandatory flour fortification, but also recommendations in the US that all women of childbearing age, not just those planning pregnancy, consume daily 0.4 mg of folate over and above that in a natural diet [11].

## Obstetric Implications

These tantalizing findings add further impetus to the study of preconceptional factors and interventions that impact on duration of pregnancy. The ultimate evidence as to whether folic acid prevents spontaneous preterm birth will require a randomized controlled trial, but conducting such a trial may prove challenging on several fronts. First, there are robust reasons to encourage all women to take folic acid. Second, one third of the world already has mandatory folic acid fortification [11]. Third are the ethical difficulties with a control group, although these might be surmounted in geographical areas where prepregnancy supplementation is not yet supported. One practical way forward would be a randomized controlled trial of ongoing folic acid supplementation in mothers with a previous very preterm birth, ideally with high-dose and low-dose arms compared to standard care to dissect out dose versus duration effects.

## Public Health Policy: Too Little, Too Late?

There is increasing evidence that recommended supplementation levels are inadequate to optimize pregnancy outcome. Studies from North and South America show that low-level fortification of flour prevents at most only 40% of NTDs, because such fortification provides only a quarter of the recommended daily intake [16],[17]. Bukowski and colleagues' study confirms that fewer than 20% of women follow recommendations for additional folate, while in settings without mandatory fortification of flour, such as most of Europe, as few as 5% of women take the recommended 400 µg dose in the three months prior to conception [10]. Higher daily doses result in higher folate levels, and there is a continuous dose–response relationship between early pregnancy folate levels and NTD prevention [18]. Compelling arguments have been made to increase mandatory flour fortification levels 2–4 fold and pre-pregnancy folic acid tablets to 4–5 mg per day, aiming to prevent around 85% of NTDs [17]. There is little downside, now that earlier concerns about folic acid unmasking vitamin B12 deficiency appear resolved, and the evidence on whether folate supplementation increases twinning remains inconclusive [19],[20].

Does Bukowski and colleagues' study provide additional impetus for an increase in the recommended dose of folic acid [19]? No, that would be premature in the absence of intervention studies to substantiate folic acid reducing very preterm birth. This is particularly important given the experience with cardiovascular disease, where epidemiological evidence suggested protective effects of folic acid supplementation that were not borne out in subsequent randomized trials [21]. In the interim, super-supplementation can be justified entirely on the basis that it would double the number of NTDs prevented.

## Abbreviations

NTD: neural tube defect
